# The novel norcantharidin derivative DCZ5417 suppresses multiple myeloma progression by targeting the TRIP13–MAPK–YWHAE signaling pathway

**DOI:** 10.1186/s12967-023-04739-7

**Published:** 2023-11-27

**Authors:** Yingcong Wang, Sanfeng Dong, Ke Hu, Li Xu, Qilin Feng, Bo Li, Guangli Wang, Gege Chen, Bibo Zhang, Xinyan Jia, Zhijian Xu, Xuejie Gao, Hui Zhang, Yongsheng Xie, Meiling Lu, Shuaikang Chang, Dongliang Song, Xiaosong Wu, Qi Jia, Huabin Zhu, Jinfeng Zhou, Weiliang Zhu, Jumei Shi

**Affiliations:** 1 Department of Hematology, Shanghai East Hospital, Tongji University School of Medicine, Shanghai, 200120 China; 2 CAS Key Laboratory of Receptor Research, State Key Laboratory of Drug Research; Drug Discovery and Design Center, Shanghai Institute of Materia Medica, Chinese Academy of Sciences, Shanghai, 201203 China; 3 Department of Hematology, Shanghai Tenth People’s Hospital, Tongji University School of Medicine, Shanghai, 200072 China; 4 Department of Hematology, The Affiliated People’s Hospital of Ningbo University, Ningbo, 315000 China; 5 Shanghai University of Traditional Chinese Medicine, Shanghai, 201203 China

**Keywords:** Norcantharidin, Derivative, DCZ5417, TRIP13, Anti-myeloma, MAPK

## Abstract

**Background:**

Multiple myeloma (MM), an incurable disease owing to drug resistance, requires safe and effective therapies. Norcantharidin (NCTD), an active ingredient in traditional Chinese medicines, possesses activity against different cancers. However, its toxicity and narrow treatment window limit its clinical application. In this study, we synthesized a series of derivatives of NCTD to address this. Among these compounds, DCZ5417 demonstrated the greatest anti-MM effect and fewest side effects. Its anti-myeloma effects and  the mechanism were further tested.

**Methods:**

Molecular docking, pull-down, surface plasmon resonance-binding, cellular thermal shift, and ATPase assays were used to study the targets of DCZ5417. Bioinformatic, genetic, and pharmacological approaches were used to elucidate the mechanisms associated with DCZ5417 activity.

**Results:**

We confirmed a highly potent interaction between DCZ5417 and TRIP13. DCZ5417 inhibited the ATPase activity of TRIP13, and its anti-MM activity was found to depend on TRIP13. A mechanistic study verified that DCZ5417 suppressed cell proliferation by targeting TRIP13, disturbing the TRIP13/YWHAE complex and inhibiting the ERK/MAPK signaling axis. DCZ5417 also showed a combined lethal effect with traditional anti-MM drugs. Furthermore, the tumor growth-inhibitory effect of DCZ5417 was demonstrated using in vivo tumor xenograft models.

**Conclusions:**

DCZ5417 suppresses MM progression in vitro, in vivo, and in primary cells from drug-resistant patients, affecting cell proliferation by targeting TRIP13, destroying the TRIP13/YWHAE complex, and inhibiting ERK/MAPK signaling. These results imply a new and effective therapeutic strategy for MM treatment.

**Supplementary Information:**

The online version contains supplementary material available at 10.1186/s12967-023-04739-7.

## Introduction

Multiple myeloma (MM), a cancer of terminally differentiated plasma cells, is the second most common hematological malignancy, and it has a high incidence rate, poor prognosis, and high rate of relapse, with drug resistance often observed [1, 2]. A study found that the incidence was increasing annually. Moreover, Western Europe, Australia, the United States, and other developed countries have significantly higher incidences than Asian countries [3, 4]. The clinical manifestations in patients usually include renal insufficiency, anemia, hypercalcemia, and pathological bone disease [5, 6]. Further, monoclonal proteins secreted by the malignant plasma cells are the most important signs of MM [5, 7]. Clinical manifestations are usually driven by monoclonal proteins, malignant cells, or cytokines secreted by malignant cells [5]. Some progress has been made in MM treatment with the application of high-dose chemotherapy, new targeted drugs, hematopoietic stem cell transplantation, and cell therapy [7–9]. However, MM is still incurable, and its treatment still faces great challenges. Scientists have thus been attempting to develop effective and safe drugs for several years.

Norcantharidin (NCTD) is a demethylated derivative of cantharidin, an active ingredient in traditional Chinese medicines [10], and it has cytotoxic effects on liver, lung, breast, bladder, gastric, and esophageal cancer [11, 12]. NCTD exerts its cytotoxic effects by inhibiting cell proliferation and inducing apoptosis [12, 13]. However, its clinical applications are limited because of its narrow treatment window and toxicity.

Thyroid hormone receptor-interacting protein 13 (TRIP13) is a member of the AAA ATPase family [14–16], and accumulating evidence indicates that it is overexpressed in multiple cancers and is associated with cancer progression, poor survival, and drug resistance [14]. We previously found that TRIP13 impaired mitotic checkpoint surveillance and induced cell growth and drug resistance in MM [17]. Mechanistically, its biological function was found to be mediated by crosstalk among many signaling pathways. For example, TRIP13 plays an oncogenic role in glioblastoma via the FBXW7/c-MYC pathway [18] and induces hepatocellular carcinoma (HCC) cell migration, invasion, and metastasis through AKT/mTOR signaling via and interaction with ACTN4 [19]. It further promotes the proliferative and invasive abilities of lung cancer cells by activating the Wnt signaling pathway [20]. TRIP13 regulates EMT progression via the Notch signaling pathway [21]. TRIP13 also targets nonhomologous end-joining (NHEJ) to overcome treatment failure in squamous cell carcinoma of the head and neck (SCCHN) [22]. Moreover, it plays a critical role in B-cell lymphoma and MM by regulating the deubiquitination of critical oncogenic (NEK2) and tumor suppressor (PTEN and p53) proteins [23]. However, further studies are required to elucidate the mechanism of TRIP13-induced cancer progression. In this study, we revealed that TRIP13 was involved in activating the ERK/MAPK signaling pathway.

Based on the structure of NCTD, we attempted to modify it into a more active and less toxic compound. We designed and synthesized a series of NCTD derivatives and evaluated their anti-MM effects. DCZ5417 exhibited the strongest anti-MM effect. Therefore, we further tested its anti-myeloma activity and drug target validation, and explored the mechanism underlying its anti-myeloma effects. Mechanistically, DCZ5417 inhibited the ERK/MARK signaling pathway by targeting TRIP13 and destroying the TRIP13/YWHAE complex.

## Materials and methods

### Cell lines

ARP-1, OCI-MY5, RPMI-8226, RPMI-8226/R5, H929R-CAR and H929R-Bor cells were provided by Dr. Wen Zhou (Central South University, China). HEK293T cells were commercially obtained from American Type Culture Collection (ATCC). Cell lines were certificated by short tandem repeat analysis. Cells were maintained in RPMI-1640 medium (Gibco, Carlsbad, CA, USA) containing 10%FBS and 1% penicillin–streptomycin. HEK293T cells were maintained in DMEM containing with 10% FBS and 1% penicillin–streptomycin.

### Patient samples

Bone marrow samples were obtained from patients with MM after obtaining written informed consent at the Department of Hematology Shanghai Tenth People's Hospital (Shanghai, China). The protocol for collection and usage of clinical samples was approved by the Shanghai Tenth People’s Hospital Ethics Committee.

### Reagents and antibody

DCZ5417, DCZ5419, DCZ5524 and DCZ5530 were synthesized by Shanghai Institute of Materia Medica, Chinese Academy of Sciences, Shanghai, China. Antibodies for TRIP13, YWHAE, Bax, Bcl-xl, Caspase-3 and Caspase-8 were purchased from Abcam; p-ERK, ERK, CDK4, CDK6, Cyclin D1, a-tubulin, TUNEL, Ki-67 and β-actin were from Cell Signaling Technology. Annexin V-FITC and propidium iodide (PI) detection kit was purchased from BD.

### Acute toxicity study

The acute toxicity test was carried out strictly according to the Organization for Economic Cooperation and Development (OECD) guideline 423 (OECD 423, 2001). Before intraperitoneal injection, 21 healthy female mice (7 weeks old) were randomly divided into three experimental groups, namely, DCZ5417, NCTD 50 mg/kg i.p dose groups or vehicle 50 mg/kg i.p dose group. There were seven mice in each group. After administration, all mice were observed for mortality and changes after treatment. Signs of toxicity and mortality were also recorded. All mice were anaesthetized with chloral hydrate, and the organ (liver, kidney) of each mouse were collected.

### The metabolic stability assays

Microsomes in 0.1 M TRIS buffer pH 7.4 (final concentration 0.33 mg/mL), co-factor MgCl2 (final concentration 5 mM) and tested compound (final concentration 0.1 μM, co-solvent (0.01% DMSO) and 0.005% Bovin serum albumin (BSA)) were incubated at 37 °C for 10 min. The reaction was started by the addition of NADPH (final concentration 1 mM). Aliquots were sampled at 0, 5, 15, 30 and 60 min respectively and methanol (cold in wet ice) was added to terminate the reaction. After centrifugation (4000 rpm, 5 min), samples were then analyzed by LC–MS/MS.

### Pull-down assay

Pull-down assay was performed as described previously [24]. Briefly, the cell lysate was then incubated with 20 μmol/L DCZ5417-biotin or biotin in the presence of neutrAvidin agarose resins, and analyzed by immunoblotting.

### Surface plasmon resonance (SPR)

Pull-down assay was performed as described previously [24]. Briefly, TRIP13 protein was incubated with indicated concentrations of DCZ5417.

### Cellular thermal shift assay (CETSA)

CETSA was performed as described previously [25]. Briefly, cells were incubated with DCZ5417 or DMSO for 6 h. Then cultured cells were harvested, lysed, and analyzed by immunoblotting.

### Protein purification and ATPase assays

His-tagged TRIP-13 protein was expressed in E. coli at 16 °C for 20 h, then cells were harvested by centrifugation and fully lysed by ultrasound in lysis buffer (50 mM Tris–HCl, pH7.5, 500 mM NaCl, 10% glycerol). The supernatant lysate was purified by Ni2+-affinity and tag was then cleaved. Further, through protein chromatography column and ion-exchange column, excess proteins were removed and high-purity protein was obtained. According to the protein peak location, the sample was detected by SDS-PAGE and Coomassie was used to confirm the protein up to 95% purity. The target protein was collected, concentrated and then stored at − 80 °C.

Protein was diluted to 1 μmol/L using buffer containing 25 mM Tris–HCl pH 7.5, 200 mM NaCl, 20 mM MgCl_2_, 1 mM DTT, 5% glycerol and 0.05% Tween. Then TRIP-13 protein was pre-incubated with different concentrations of DCZ5417 for 30 min at 37 °C in advance. ATP standard was 1:1 added to compound-protein mixture until its final concentration was 100 μmol/L. The whole reaction system carried on for 1 h and then the subsequent operations were carried out according to ATPase assay kit protocol (Promega).

### Cell viability assay

Cell viability assay was performed as described previously [25]. Briefly, cells were seeded in triplicate in 96-well plates and then treated with DCZ5417. Cell viability was measured using the Cell Counting Kit (CCK)-8 assays.

### Apoptosis assay

Apoptosis assay was performed as described previously [24]. Briefly, cells were treated with or without DCZ5417. Then, cells were collected and stained with Annexin-V/PI, and then detected via flow cytometry.

### Coimmunoprecipitation (Co-IP)

Cell viability assay was performed as described previously [24]. Briefly, H929 cells were harvested and then lysed with 300 μL IP lysis buffer. Cell lysis were incubated with 30 μL Protein A/G agarose beads and specific antibodies on a rotator at 4 °C overnight. And then the pull-down complex was collected and analyzed by immunoblotting.

### Gene knockout

Knockout cells were generated using lentivirus-mediated CRISPR/Cas9 technology as described previously [24]. The single guided RNA (sgRNA) sequences targeting human TRIP13 was TGAGTAGCTTTCTAACACTC. Gene knockdown of YWHAE was generated using shRNA. Lentivirus packaging method was described previously [25]. Target sequence of YWHAE was GCTGACAGTTGAAGAAAGAAA.

### RNA-sequencing

Total RNA was extracted using the RNA-Quick purification kit (ES Science, Shanghai, China) and preserved using TRIZOL (Ambion, Austin, TX, USA), which was sent to OE biotech Co., Ltd. (Shanghai, China) for RNA-Seq analysis.

### Gene expression analysis using publicly available data sets

The Kaplan–Meier analysis of OS was performed using the database with GSE2658 and GSE57317. Gene expression profiles were performed using the Gene Expression Omnibus data sets (GSE13591). Gene expression analysis was performed using Partek Genomic Suite 6.6 with fold changes calculated as relative changes compared to normal plasma cells. Fold-changes of at least twofold with a *P* < 0.05 and an FDR < 0.05 were considered for further analysis.

### Tumor xenograft models

Tumor xenograft models were performed as described previously [24]. Briefly, human H929 cells (1X10^6^) were subcutaneously injected into the upper flank region of the nude mice. Then mice were randomly assigned to three groups: control, NCTD (15 mg/kg) and DCZ5417 (15 mg/kg). For survival assay, nude mice (6 weeks old) were injected subcutaneously in the right flank with 1X10^6^ H929 cells in a volume of 0.1 mL. After tumor growth of mice, mice were randomly assigned to three groups: control, DCZ5417 and NCTD. Mice were then administered with or without 15 mg/kg vehicle, DCZ5417 or NCTD via intraperitoneal injection. The endpoint of the experiment is that the mice sacrifice, or that tumor volume reached 2000 mm^3^. All mice were euthanized at the end of the experiment and then tumors were photographed. All animal studies were approved by the Institutional Review Board of Shanghai Tenth People’s Hospital.

### Statistical analysis

Statistical analyses were performed using GraphPad Prism 8. Data are expressed as means ± SD. The *P* values were designated exactly as shown in the figures as follows: *P < 0.05; **P < 0.01; ***P < 0.005; ****P < 0.001; N.S. not statistically significant (*P* ≥ 0.05). The log-rank test was used for survival curves. The combination index (CI) values were calculated by median dose effect analysis using commercially available software (CalcuSyn; Biosoft). All tests of statistical significance were two sided.

## Results

### Development of a highly safe and potent derivative of NCTD

**Fig. 1:**
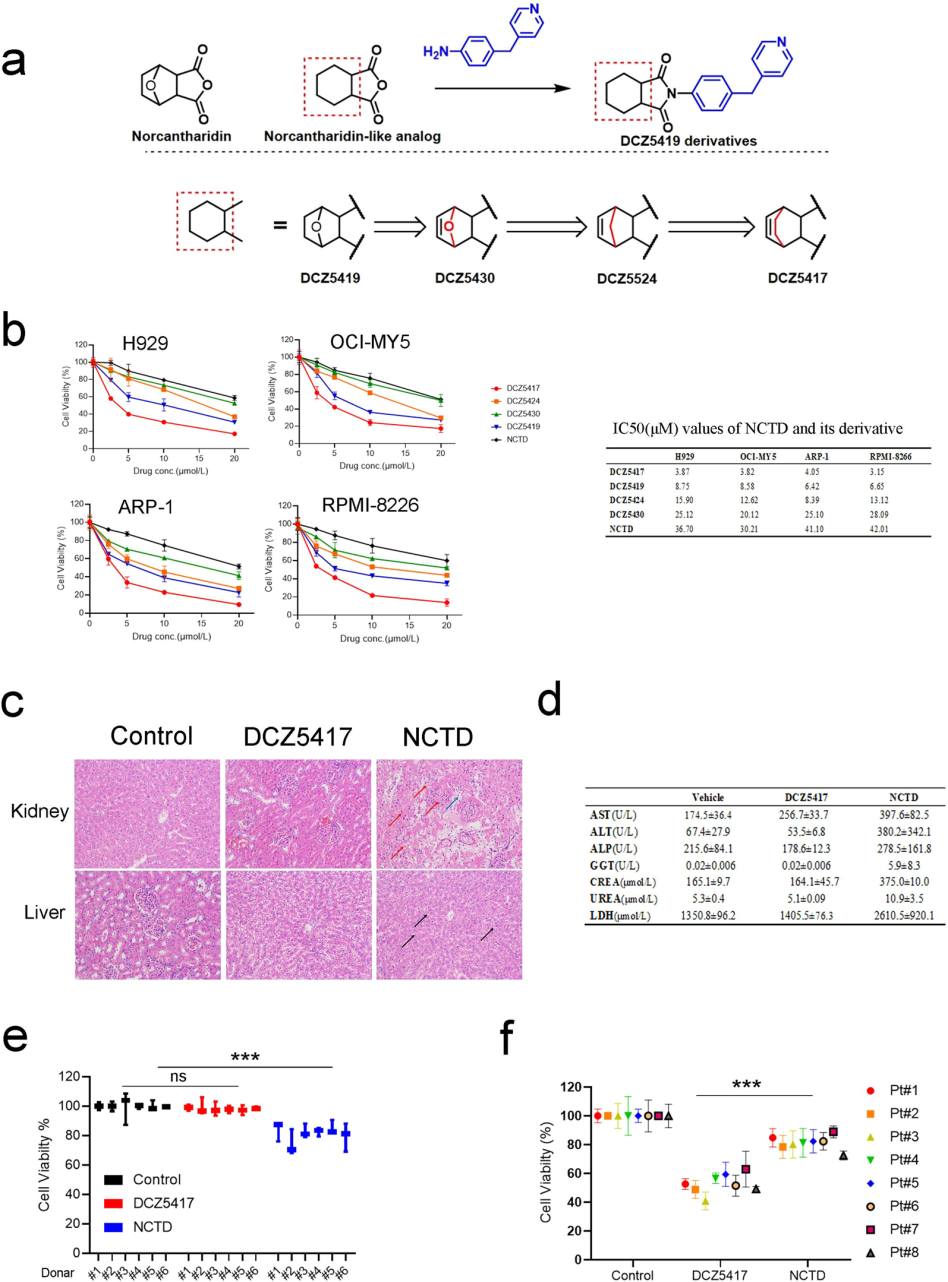
Development for a better effect and lower toxicity derivative of NCTD. **a** Derivative modification process of NCTD and structures of four different derivatives, DCZ5419, DCZ5430, DCZ5524 and DCZ5417. **b** Cell viability of MM cells with DCZ5419, DCZ5430, DCZ5524 or DCZ5417 treatment for 72 h at the indicated concentrations. IC50 (µmol/L) values of DCZ5419, DCZ5430, DCZ5524 and DCZ5417 against MM cells. IC50 values derived from GraphPad Prism are presented as mean values from three independent experiments. **c** Rats were treated with or without 50 mg/kg DCZ5417, NCTD or vehicle, respectively (6/group). H&E staining of the control, NCTD and DCZ5417-treated liver and kidney tissues. **d** Rats were treated with 15 mg/kg DCZ5417, NCTD or vehicle, respectively. Orbital blood was collected, then clinical biochemical indicators were examined. AST (Aspartate transaminase), ATL (Alanine aminotransferase), ALP (Alkaline phosphatase), GGT (Glutamine transpeptidase), CREA(Creatinine), LDH (Lactate dehydrogenase). **e** Normal PBMCs from healthy donors (PBMCs#1–PBMCs#6) were treated with 20 µmol/L DCZ5417 or NCTD for 48 h and then cell viability was analyzed. **f** Cell viability in cells from MM patients were evaluated after 10 µmol/L DCZ5417 or NCTD treatment for 48 h. Pt# representees patient number. Data are presented as the means ± SD of 3 independent experiments. *P < 0.05, **P < 0.01 and ***P < 0.001 versus the control group

NCTD has been clinically used as an effective oncology drug in China for several years. It is a demethylated analog of cantharidin with toxicity and side effects. To develop a more potent NCTD derivative with fewer side effects, we introduced 4-(4-pyridylmethyl) aniline, which has been proven a privileged skeleton, into the anhydride group of NCTD to obtain the compound DCZ5419 (Fig. 1a). However, the CCK-8 results indicated that the ability of the lead compound to inhibit cell growth was unsatisfactory. Thus, a 5,6-dehydronocantharidin nucleus containing the compound (DCZ5430) and its bioisosteres (DCZ5524) was also synthesized, and moderate inhibitory effects were observed (Fig. 1a and b). Changing the aliphatic endocyclic portion into a homologous structure (DCZ5417) resulted in the best in vitro potency (Fig. 1a and b). After structural optimization, the IC_50_ values of DCZ5417 were nearly ten fold higher than those of NCTD in MM cells (Fig. 1b). An in vivo acute toxicity study was further conducted to compare the toxicities and side effects of DCZ5417 and NCTD. For this, we administered 50 mg/kg DC5417 or NCTD to rats via an intraperitoneal injection. The livers and kidneys of rats were then compared between the groups. DCZ5417 was not significantly toxic, as the hepatocytes of treated animals were arranged regularly without obvious lesions. Moreover, the glomeruli were evenly distributed, and there was no obvious proliferation in the interstitium. No obvious inflammatory changes were observed in the kidneys (Fig. 1c). However, liver and kidney functions in the NCTD group were impaired. Specifically, the granular degeneration of hepatocytes, loose cytoplasm, and light staining (black arrow) were observed in the NCTD group. Extensive necrosis and structural disorders were also observed in the NCTD group. Further, the original structures of the renal tubules and glomeruli disappeared, indicating the presence of unstructured eosinophils. Necrotizing cell fragments were widely observed (red arrow), and lymphocyte infiltration was observed (blue arrow) in the NCTD group (Fig. 1c and Additional file 1: Figure S1a). Biochemical indicators further showed that DCZ5417 was safer than NCTD (Fig. 1d). Furthermore, peripheral blood mononuclear cells (PBMCs) were treated with DCZ5417 or NCTD, and the data showed that DCZ5417 had no significant toxicity towards healthy human PBMCs, whereas NCTD inhibited the viability of these cells by approximately 20% (Fig. 1e and Additional file 1: Figure S1b). These data indicate that DCZ5417 is safer and less toxic than NCTD. To further compare the anti-myeloma activities of the two compounds, we examined their effect on primary CD138^+^ cells from patients treated with DCZ5417 or NCTD. As shown in Fig. 1f, DCZ5417 had a greater inhibitory effect on cell viability than NCTD. Moreover, the metabolic stability of DCZ5417 in human, rat and mouse liver microsomes was determined, which showed good in vitro half-life (Thalf) with human liver microsome (66.8 min) (Additional file 1: Figure S1c). Thus, we identified a novel derivative of NCTD, named DCZ5417, with a stronger inhibitory effect, less toxicity, and better safety and efficacy than NCTD, which could be a better lead compound for anti-MM drug development.

### DCZ5417 exhibits potent anticancer activity

**Fig. 2:**
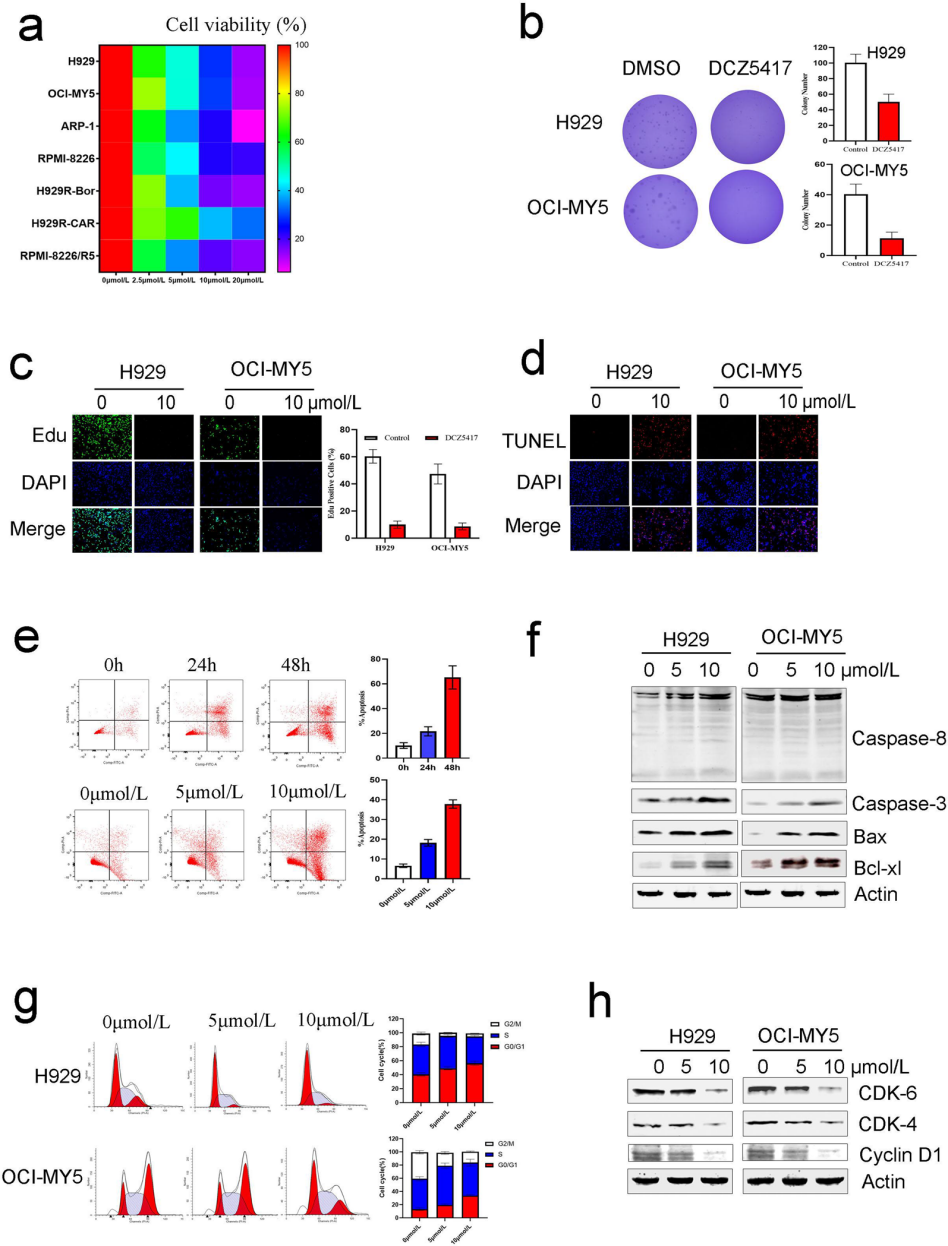
DCZ5417 inhibits multiple myeloma. **a** Indicated MM cell lines were treated with vehicle or DCZ5417 at the indicated concentrations for 72 h. Then the cell viability was determined by CCK-8 assay. Cell viability data are presented as the means of 3 independent experiments in a Heatmap. **b** Soft agar colony formation of H929 and OCI-MY5 cells were tested after DCZ5417 or DMSO treatment. Quantification of the colony numbers in the left. *P < 0.05. **c** H929 and OCI-MY5 cells were exposed to 0 and 10 µmol/L DCZ5417 for 48 h, Edu assay was carried out. Statistics of Edu-positive cells in the left. **d** Representative fluorescent images of typical apoptotic cells evaluated by TUNEL staining (red) after 10 µmol/L DCZ5417 treatment for 24 h. DAPI was used as a nuclear stain (blue). **e** Flow cytometry evaluation of Annexin-V-positive apoptotic cells in DCZ5417-treated H929 cells. Representative results of triplicate experiments were shown. **f** Immunoblotting was used to analyze the expression levels of Caspase-3, Caspase-8, Bax, and Bcl-xL. **g** Cell-cycle analysis of DCZ5417 (0, 5, and 10 µmol/L, 24 h)-treated H929 and OCI-MY5 cells. **h** Effects of DCZ5417 treatment on CDK4, CDK6 and Cyclin D1 expression in cells. Data are presented as the means ± SD of 3 independent experiments. *P < 0.05, **P < 0.01 and ***P < 0.001 versus the control group

To further investigate the effects of DCZ5417 on MM cells, the H929, OCI-MY5, ARP-1, RPMI-8226, H929R-Bor, H929R-CAR, and RPMI-8226/R5 cell lines were selected for this study. DCZ5417 significantly inhibited the viability of MM cells (*P* < 0.05) (Fig. 2a and Additional file 1: Figure S2a). We then tested whether DCZ5417 could regulate MM cell colony formation, and for this, soft-agar clonogenic assays were performed using H929 and OCI-MY5 cells. As shown in Fig. 2b, DCZ5417 significantly inhibited colony formation (*P* < 0.05). To examine whether DCZ5417 affected DNA synthesis, an EdU assay was performed using H929 and OCI-MY5 cells. Here, the percentage of EdU-positive cells was significantly decreased upon treatment with DCZ5417 (Fig. 2c). This indicates that DCZ5417 inhibits DNA synthesis in MM cells. Compared to that in the controls, the percentage of TUNEL-positive cells after DCZ5417 treatment was increased (Fig. 2d). To further assess the anti-myeloma activity of DCZ5417, H929, OCI-MY5, and H929R cells were treated with DCZ5417 (0–40 μmol/L) for 24, 48, and 72 h. DCZ5417 significantly decreased the cell viability in a time- and dose-dependent manner (Additional file 1: Figure S2b). Next, we examined whether DCZ5417 could induce apoptosis. MM cells were treated with various concentrations of DCZ5417, which induced apoptosis in a dose- and time-dependent manner (Fig. 2e). The effects of DCZ5417 on the expression of caspase-related proteins were also evaluated. Compared with those in the control, Caspase-8, Caspase-3, Bcl-xl, and Bax activities were increased in a dose-dependent manner in cells treated with DCZ5417 (Fig. 2f). In addition to apoptosis, the effect of DCZ5417 on cell cycle progression was evaluated. As shown in Fig. 2g, DCZ5417 treatment induced the significant accumulation of MM cells in the G0–G1 phase. The expression of cyclin-related proteins was also examined in cells treated with or without DCZ5417. We observed a marked dose-dependent decrease in Cyclin D1, CDK4, and CDK6 expression in cells treated with DCZ5417 compared to that in the control cells (Fig. 2h). These results indicated that DCZ5417 induced cell apoptosis and arrested cell cycle progression at the G0–G1 phase.

### DCZ5417 binds TRIP13 and inhibits its activity

**Fig. 3:**
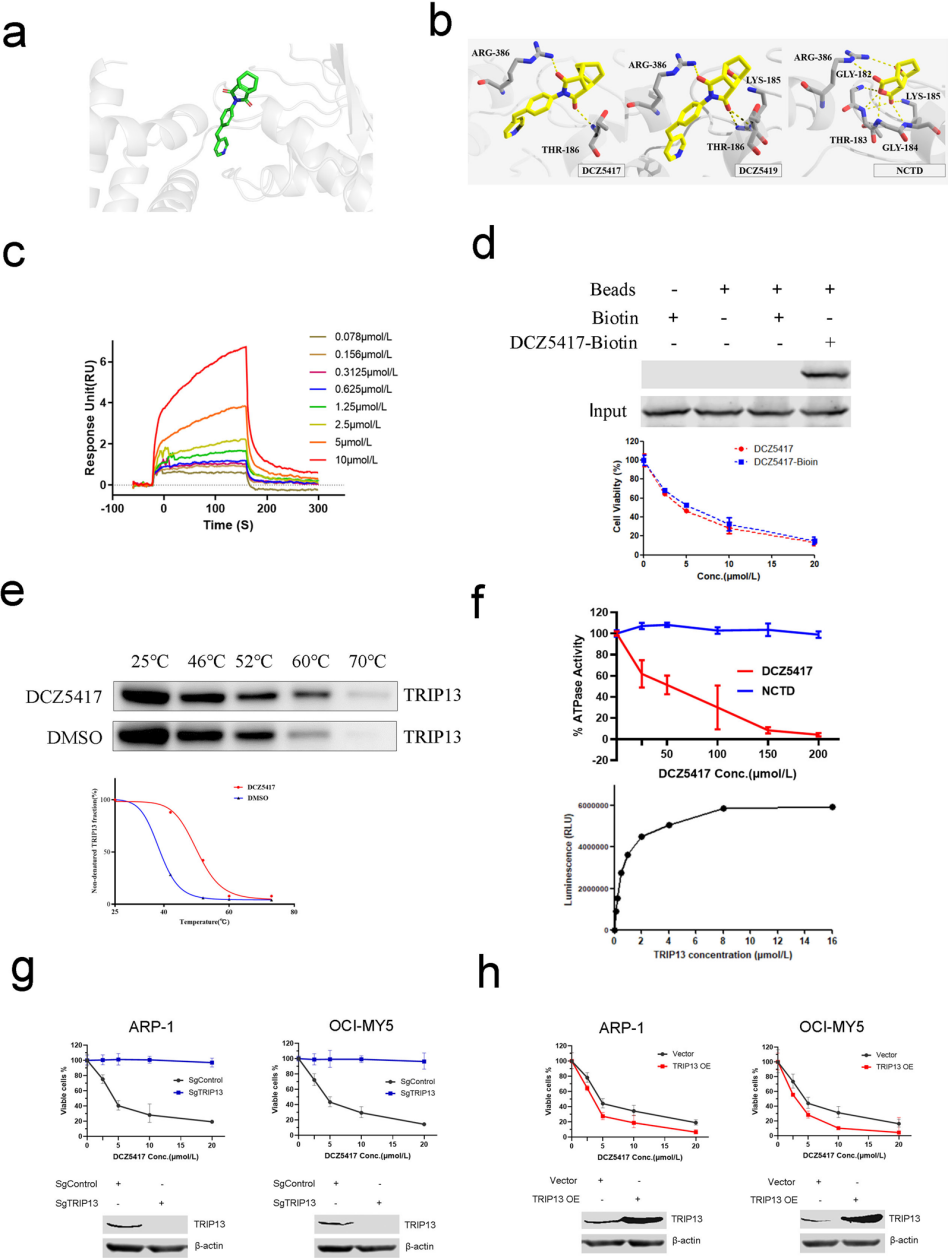
DCZ5417 specifically binds and inhibits TRIP13 in Cells. **a** The structure of DCZ5417 (green) and its binding mode to TRIP13 (gray) as determined by molecular docking. **b** The binding mode of DCZ5417, DCZ5419 and NCTD with TRIP13 determined by molecular docking. **c** SPR biosensor was used to detect the binding of DCZ5417 to TRIP13. **d** A pull-down assay was used to detect the binding of DCZ5417-biotin to TRIP13. Anti-MM activity of DCZ5417 and DCZ5417-biotin was compared (below). **e** Cellular thermal shift assay to examine interactions of DCZ5417 (10 µmol/L) with TRIP13. **f** Relative ATPase activity was examined after DCZ5417 and NCTD treatment by ADP-Glo™ Kinase Assay. **g** The viability of MM cells transfected with empty vector or TRIP13-sgRNA with DCZ5417 treatment (0, 2.5, 5, 10 and 20 µmol/L, 48 h) was analyzed by a CCK-8 assay. SgControl represents nontarget scramble-transfected cells. TRIP13 sgRNA represents TRIP13-silenced cells. The result is expressed as means SD of three independent experiments. **h** CCK-8 assay was performed on TRIP13 OE cells or empty vector-transfected cells. Immunoblotting was used to examine protein level of TRIP13 in TRIP13 OE and Vector cells. Vector represents nontarget scramble–transfected cells. TRIP13 OE represents overexpression of TRIP13 in cells. Data are presented as the means ± SD of 3 independent experiments. *P < 0.05, **P < 0.01 and ***P < 0.001 versus the control group

Virtual docking analysis was performed to study the targets of DCZ5417, and the results showed that DCZ5417 targeted TRIP13 (Fig. 3a). In further, we carried out the molecular docking study to compare the binding mode of DCZ5417, DCZ5419 and NCTD with TRIP13 (Fig. 1b). It indicated that the DCZ5417 showed a slightly better docking score compared with DCZ5419 (− 8.15 kcal/mol vs − 8.08 kcal/mol). NCTD cannot bind TRIP13 very well (− 5.97 kcal/mol). ARG-386 and THR-186 were two key residues that can form the hydrogen bonds with two carbonyl groups respectively. Besides, the α-amino group of LYS-185 can also form a hydrogen bond with DCZ5419 (Fig. 3b). We then performed a series of assays to determine whether DCZ5417 targeted this protein. An SPR assay was used to measure the interaction between TRIP13 and DCZ5417 (Fig. 3c). Furthermore, an affinity pull-down target verification system was used to validate interactions between DCZ5417 and TRIP13. To perform the affinity assay, DCZ5417 was conjugated with biotin, named DCZ5417-biotin. Cell viability was also tested to determine whether biotin conjugation influenced the activity of DCZ5417, and DCZ5417-biotin had no significant effect on the anti-myeloma activity of DCZ5417 (Fig. 3d, below). The pull-down assay showed that, compared to that in the control, the addition of DCZ5417-biotin to the cell lysate resulted in the downregulation of endogenous TRIP13 expression (Fig. 3d, up). These data suggest that DCZ5417 binds TRIP13. We further investigated the binding activity of DCZ5417 to TRIP13 at the cellular level using CETSA. DCZ5417 increased the thermal stability of TRIP13 (Fig. 3e), suggesting a specific physical interaction between DCZ5417 and TRIP13. Furthermore, we examined whether DCZ5417 would inhibit the ATPase activity of TRIP13. First, we selected the appropriate concentration of TRIP13 (Fig. 3f, below). According the result, ATPase activity was measured with (0–200 μmol/L) DCZ5417 or NCTD treatment with 1 μmol/L TRIP13 (Fig. 3f, up), and the data showed that DCZ5417 inhibited ATPase activity. However, NCTD had no significant effect on ATPase activity.

To determine whether the anti-myeloma activity of DCZ5417 depended on TRIP13, stable TRIP13-knockout and TRIP13-overexpressing MM cell lines were established. The treatment of sgTRIP13 cells with DCZ5417 led to the loss of sensitivity to DCZ5417 compared to that with sgControl-transfected wild-type cells (Fig. 3g). However, the overexpression of TRIP13 increased sensitivity to DCZ5417 (Fig. 3h). These results suggest that the anti-myeloma activity of DCZ5417 depends on TRIP13.

### DCZ5417 inhibits MM via the ERK/MAPK signaling pathway

**Fig. 4:**
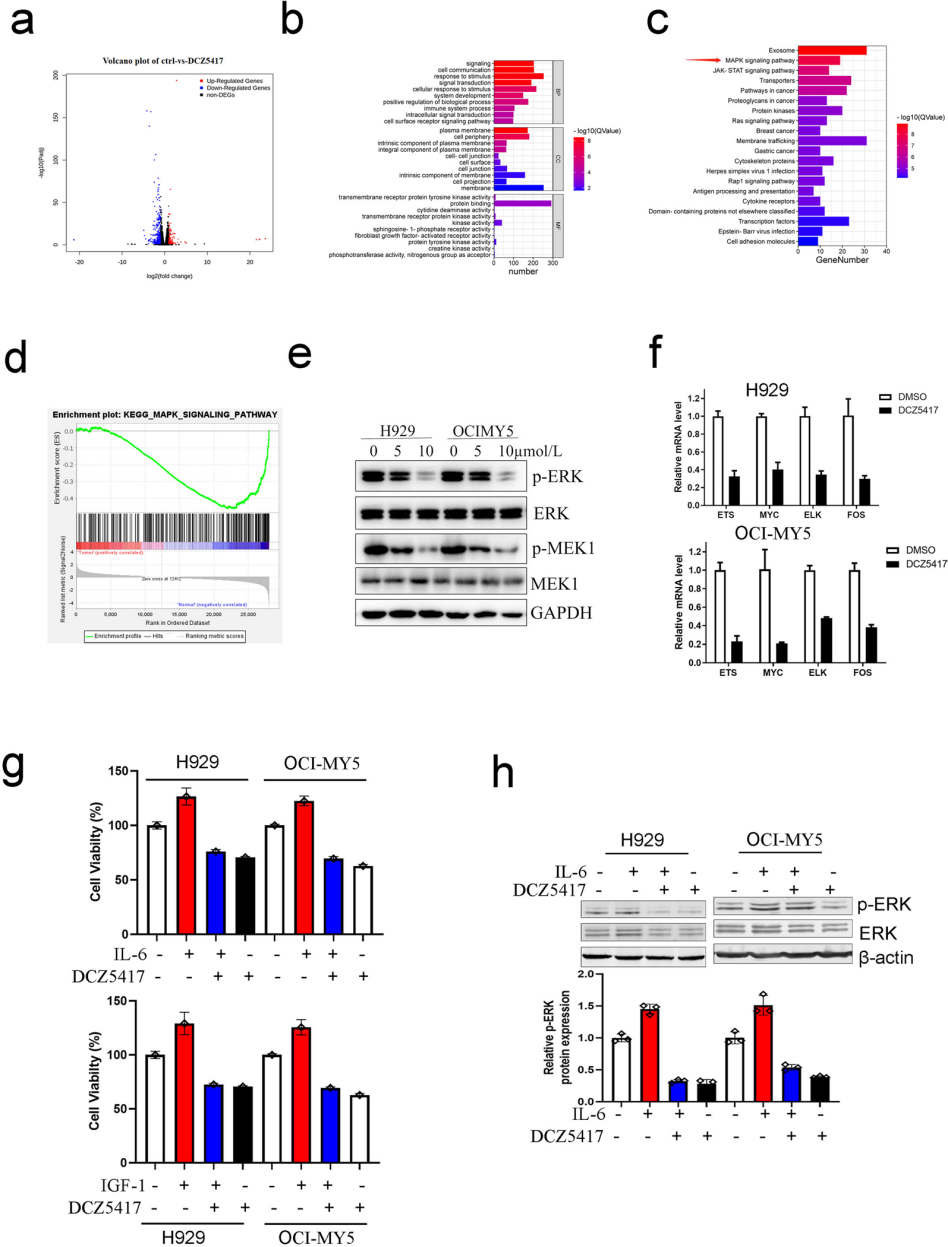
DCZ5417 inhibits MAPK signaling pathway. **a**–**d** A Gene expression profiling was performed after H929 cells were treated with 10 µmol/L DCZ5417 for 48 h. Representational and pathway analysis of differentially expressed genes in DCZ5417 or DMSO treated cells. **a** Volcano plot and bar graphs; **b** GO terms; **c** KEGG pathways. **d** Gene Set Enrichment (GSEA) analysis was conducted. An enrichment plot for MAPK pathway in cells. **e** Immunoblotting analysis of p-ERK/ERK and p-MEK/MEK in cell lysates of H929 and OCI-MY5 cells treated with indicated concentration of DCZ5417. **f** Relative mRNA expressions of ETS, MYC, ELK and FOS were examined in H929 and OCI-MY5 cells treated with DCZ5417 or DMSO. **g** Activity of DCZ5417 against H929 and OCI-MY5 cell lines cultured in the presence or absence of IL-6 and IGF-1 for 48 h. Error bars, SD. The result is expressed as means ± SD of three independent experiments. **h** H929 and OCI-MY5 cells were collected after DCZ5417 treatment with IL-6 or not, and then relative expression of p-ERK/ERK was analyzed by Immunoblotting. All results are expressed as means SD of three independent experiments. *P < 0.05, **P < 0.01 and ***P < 0.001 versus the control group

To study the mechanism underlying the anti-myeloma effect of DCZ5417, global gene expression profiling analysis was performed on H929 cells treated with DCZ5417. As shown in Fig. 4a, more than 1,900 genes were differentially expressed in cells (*P* < 0.001; Fig. 4a). Gene Ontology (GO) and Kyoto Encyclopedia of Genes and Genomes (KEGG) analyses were then performed. GO analysis revealed that signal transduction, intracellular signaling pathways, and cell surface receptor signaling pathway genes were significantly enriched, among biological processes, in cells treated DCZ5417 (Fig. 4b). KEGG analysis confirmed that the MAPK signaling pathway was significantly enriched in cells treated DCZ5417 (Fig. 4c). Gene set enrichment analyses (GSEAs) identified the MAPK signaling pathway as significantly suppressed in cells treated with DCZ5417 (Fig. 4d). To confirm the effect of DCZ5417 treatment on the MAPK pathway, the levels of MAPK signaling pathway-related proteins were examined in cells treated with DCZ5417 or DMSO. Immunoblotting showed that the phosphorylation of ERK (p-ERK) and phosphorylation of MEK (p-MEK) were decreased in a dose-dependent manner in MM cells treated with DCZ5417 (Fig. 4e). Validation through quantitative real-time PCR showed that DCZ5417 treatment significantly reduced the mRNA levels of MAPK pathway-related genes such as *ETS*, *ELK1*, *MYC*, and *FOS* in H929 and OCI-MY5 cells (Fig. 4f).

To explore whether the DCZ5417-induced suppression of cell growth was dependent on the MAPK signaling pathway, the cytokines IL-6 and IGF-I were examined. We found that IL-6 and IGF induced cell proliferation and that DCZ5417 nearly completely abrogated IL-6- and IGF-induced cell proliferation (Fig. 4g). We chose the alkylating agent melphalan as a control and found that it could not overcome IL-6- or IGF-induced cell growth (Additional file 1: Figure S2c and S2d). The increase in p-ERK induced by the MAPK signaling activator IL-6 was also abrogated by DCZ5417 (Fig. 4h). These data show that DCZ5417 suppresses cell proliferation via the MAPK/ERK signaling pathway.

### TRIP13 promotes ERK/MAPK pathway activation via YWHAE

**Fig. 5:**
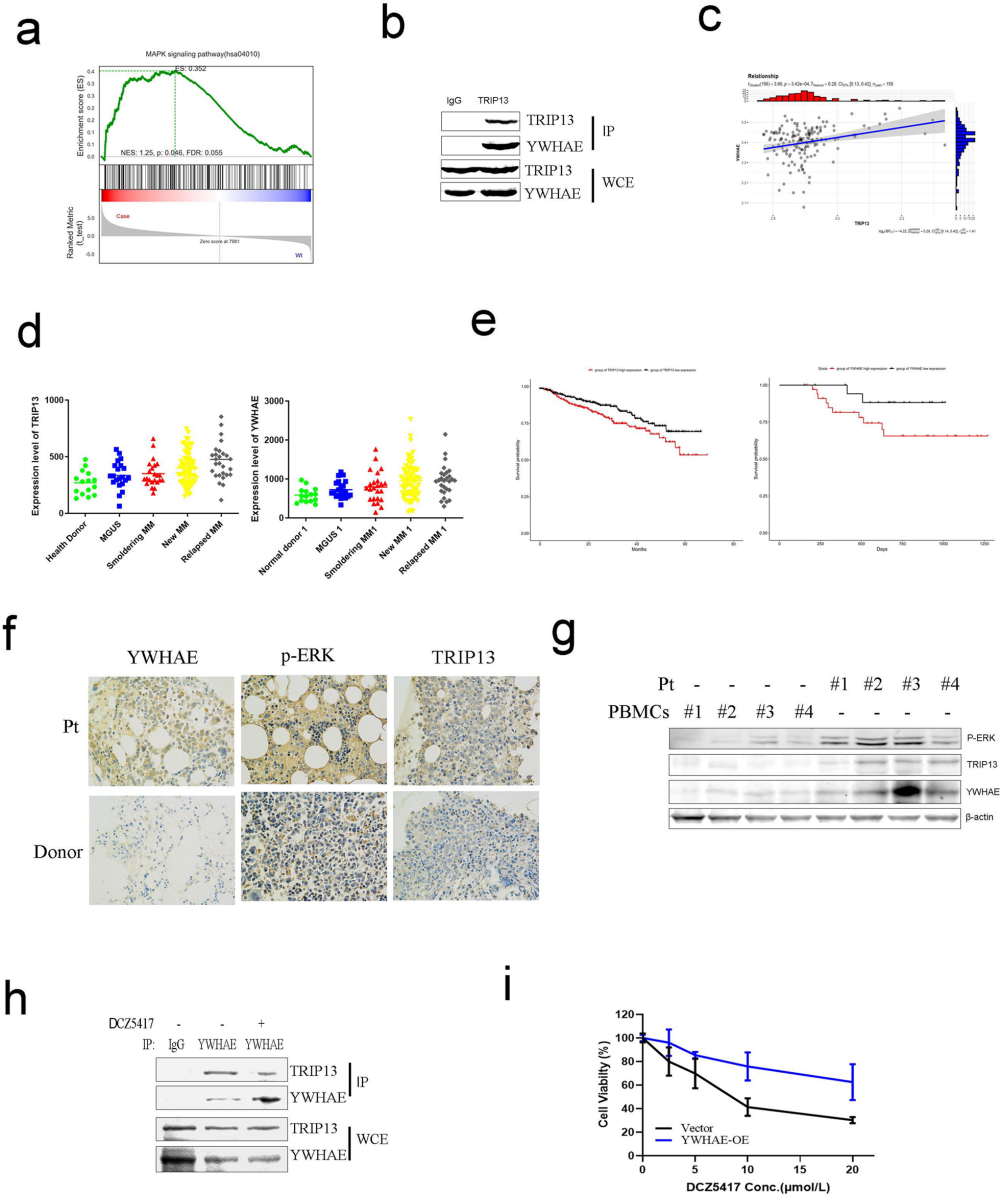
TRIP13 regulates MAPK/ERK pathway via YWHAE. **a** RNA-seq analysis of the differentially expressed transcripts in EV and TRIP13-OE ARP-1 cells (red and blue represent high and low mRNA expression levels, respectively). Case represents ARP-1 cells stably transfected with lentivirus-mediated human TRIP13-cDNA (TRIP13-OE); Wt represents ARP-1 cells stably transfected with the empty vector (EV). GSEA indicated a positive correlation between TRIP13 overexpression and MAPK signaling activation. **b** Endogenous co-immunoprecipitation was conducted with H929 cells using anti-YWHAE, followed by immunoblotting using anti-TRIP13. Anti-IgG was used as a non-specific control. **c** and **d** An Analysis of TRIP13 and YWHAE expression in publicly available MM patient data sets. Increased TRIP13 or YWHAE expression is observed in plasma cells from patients with MGUS, SMM, MM and relapsed MM than from normal healthy donors. **e** Kaplan–Meier analyses of OS about patients from TT2 (p < 0.001) and TT3 (p < 0.05) cohorts revealed inferior outcomes among the patients with high (quartiles 4) TRIP13 or YWHAE expression compared with the remaining patients with low (quartiles 1–3) TRIP13 or YWHAE expression. **f** Immunohistochemical analysis of TRIP13 and YWHAE expression (positive cells are brown) in 3 representative BM specimens derived from normal and MM patients. **g** Immunoblotting examined that protein levels of TRIP13 and YWHAE in cells from MM patients compared with that of the normal group. **h** Co-immunoprecipitation of TRIP13 and YWHAE in H929 cells with or without DCZ5417. **i** Activity of DCZ5417 against Vector or YWHAE-OE cells were examined by CCK-8 assay. All results are expressed as means SD of three independent experiments. *P < 0.05, **P < 0.01 and ***P < 0.001 versus the control group

Since DCZ5417 was found to decrease cell viability via the ERK/MAPK signaling pathway and DCZ5417 suppressed cell proliferation by targeting TRIP13, we studied the relationship between TRIP13 and the ERK/MAPK signaling pathway. To determine the role of TRIP13 in the regulation of the ERK/MAPK signaling pathway, RNA-seq analysis of TRIP13 overexpression (OE) and vector (EV) cells was performed. RNA-seq data analysis indicated that a subset of MAPK signaling-related genes was differentially expressed in cells with TRIP13 overexpression. Further, GSEA showed that the protein level of TRIP13 in MM cells was positively correlated with ERK/MAPK signaling (Fig. 5a). Public database analysis also showed that TRIP13 is positively related to the MAPK signaling pathway (Additional file 1: Figure S3a). To confirm these results, immunoblotting was performed to examine the expression of MAPK signaling-related proteins. The data indicated that, compared with those in the control, the overexpression of TRIP13 increased the protein levels of p-ERK and p-MEK, indicating that TRIP13 could activate the MAPK signaling pathway (Additional file 1: Figure S3b and S3c). To study how TRIP13 regulates the ERK/MAPK signaling pathway, liquid chromatography–tandem mass spectrometry was performed to identify potential TRIP13-interacting proteins. However, among these proteins, RAF, MEK, and ERK were not found to interact with TRIP13. However, among the top 10 proteins with the strongest binding to TRIP13, YWHAE was determined to be associated with the ERK/MAPK signaling pathway (Additional file 1: Figure S3d). YWHAE has been extensively reported to be involved in ERK/MAPK signaling-mediated cell proliferation. To confirm the interaction between TRIP13 and YWHAE, endogenous co-immunoprecipitation was performed using H929 cells. As shown in Fig. 5b, TRIP13 and YWHAE were found to be tightly bound to each other.

We next analyzed public databases widely used in oncology research to further clarify the clinical relationship between TRIP13 and YWHAE. According to the database, YWHAE is positively correlated with TRIP13 expression in MM (Fig. 5c). Additionally, the overexpression of YWHAE and TRIP13 was determined to be associated with an advanced tumor stage, and patients with plasma cell leukemia (PCL) had higher YWHAE levels (Fig. 5d). Importantly, TRIP13 and YWHAE were closely associated with the prognosis of patients with MM, and those with high TRIP13 or YWHAE expression had poor overall survival (Fig. 5e), indicating that YWHAE plays a key role in the progression of MM. These results suggest that YWHAE interacts with TRIP13 and is involved in ERK/MAPK signaling and that DCZ5417, a TRIP13 inhibitor, can inhibit this signaling in MM.

To study the clinical significance of TRIP13, YWHAE, and p-ERK in MM, the expressions of these proteins were evaluated based on the immunohistochemical staining of bone marrow biopsies, including three relapsed or refractory myeloma samples and three normal samples. The protein levels of TRIP13, YWHAE, and p-ERK were significantly higher in tumor tissues than in normal tissues (Fig. 5f). In addition, CD138^+^ cells from four patients with relapsed refractory myeloma and PBMCs from four healthy donors were collected, and the protein levels of TRIP13, YWHAE, and p-ERK were determined by performing immunoblotting. The expressions of TRIP13, YWHAE, and p-ERK in relapsed refractory patients were higher than that in normal plasma cells (Fig. 5g). These data indicated that TRIP13/YWHAE positively regulated the ERK/MAPK signaling pathway.

To study the detailed mechanism though which the ERK/MAPK signaling pathway was regulated by DCZ5417, endogenous Co-IP was performed with or without DCZ5417 treatment. Compared to that in the control, the interaction between TRIP13 and YWHAE was greatly weakened in cells treated with DCZ5417 (Fig. 5h), indicating that inhibition of the MAPK signaling pathway might be regulated by DCZ5417 through a disruption of the interaction between TRIP13 and YWHAE. To examine whether the DCZ5417-mediated suppression of proliferation was dependent on YWHAE, a stable cell line overexpressing YWHAE (YWHAE-OE) was established. Cell viability was compared between the groups (Vector and YWHAE-OE) treated with DCZ5417. As shown in Fig. 5i, DCZ5417 decreased cell viability in the control group, whereas YWHAE-OE cells were resistant to DCZ5417. This suggests that the TRIP13/YWHAE signaling axis is essential for the anti-MM activity of DCZ5417.

### DCZ5417 enhances the cytotoxicity of conventional (melphalan and lenalidomide) agents

**Fig. 6:**
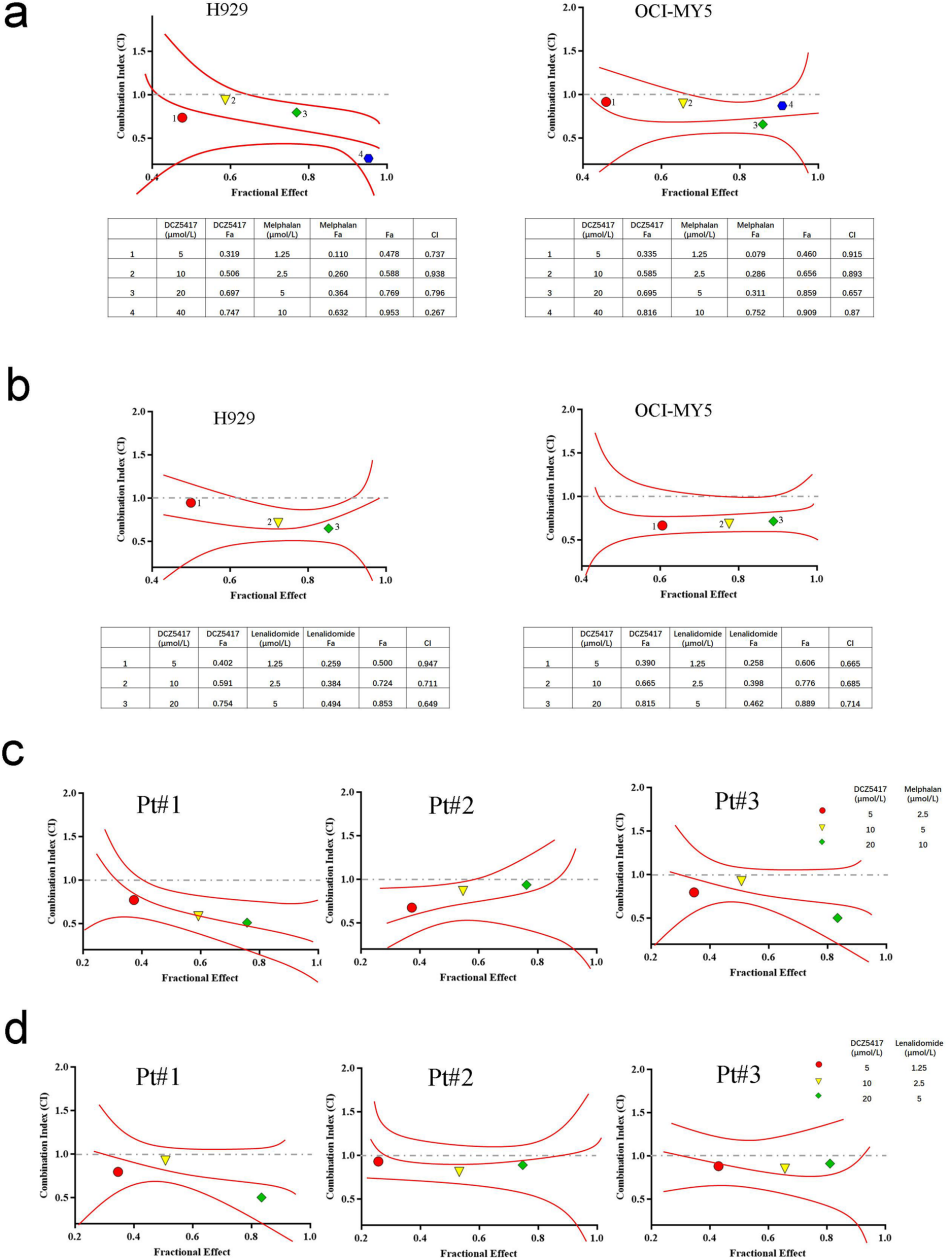
DCZ5417 in combination with melphalan or lenalidomide functions synergistically to exert cytotoxicity. **a** H929 and OCI-MY5 cells were treated with DCZ5417 (5–40 µmol/L) plus melphalan (1.25–10 µmol/L) for 48 h, which was followed by a CCK-8 assay to determine cell viability. The synergistic cytotoxic effects of DCZ5417 and melphalan are shown. CI < 1 indicated synergistic activity, as determined using CalcuSyn software. The Fa fraction represented the cells affected. **b** H929 and OCI-MY5 cells were treated with DCZ5417 (5–20 µmol/L) plus lenalidomide (1.25–5 nmol/L) for 48 h, which was followed by a CCK-8 assay to determine cell viability. **c** Purified CD138^+^ patient MM cells were inoculated in 96-well plates and treated with DCZ5417 and melphalan for 48 h. CCK-8 assay was performed to assess viability. **d** Purified CD138^+^ patient MM cells were inoculated in 96-well plates and treated with DCZ5417 and lenalidomide for 48 h. CCK-8 assay was performed to assess viability. Isobologram analysis shows the synergistic cytotoxic effect. Data are presented as the means ± SD of 3 independent experiments. *P < 0.05, **P < 0.01 and ***P < 0.001 versus the control group

To further evaluate its preclinical efficacy, we investigated the effects of DCZ5417 on cell growth when used in combination with other anti-myeloma agents. Melphalan, a DNA-damaging agent, is widely used as a preparative agent in patients with MM undergoing autologous stem cell transplantation, and the DNA repair capacity of MM cells represents an important mechanism of melphalan resistance [26]. The cytotoxicity of the combination of DCZ5417 and melphalan was examined in MM cells. Melphalan-induced growth inhibition was enhanced by increasing the DCZ5417 concentration. Calculation of the CI values using CalcuSyn software revealed synergistic effects for DCZ5417 and melphalan against MM cells (Fig. 6a). Further, cells were cultured with lenalidomide (1.25, 2.5, and 5 μmol/L) for 48 h in the presence or absence of DC5417. A CCK8 assay revealed that lenalidomide alone significantly inhibited cell growth in a dose-dependent manner, and DCZ5417 enhanced these growth-inhibitory effects in an additive manner. Importantly, a synergistic effect of DCZ5417 and lenalidomide was observed in H929 and OCI-MY5 cells (Fig. 6b).

To further validate the clinical significance of DCZ5417, we examined the effect of this combined treatment on primary CD138^+^ cells from patients with relapsed refractory myeloma. The data revealed that the combination treatment with DCZ5417 and melphalan exerted a favorable synergistic effect in these patients (Fig. 6c). At the same time, the combination treatment with DCZ5417 and lenalidomide had a synergistic effect on CD138^+^ cells from patients with myeloma (Fig. 6d). These results indicate that DCZ5417 enhances the cytotoxicity of conventional agents in MM cell lines and primary cells from patients with myeloma.

### MM xenografts are sensitive to DCZ5417

**Fig. 7:**
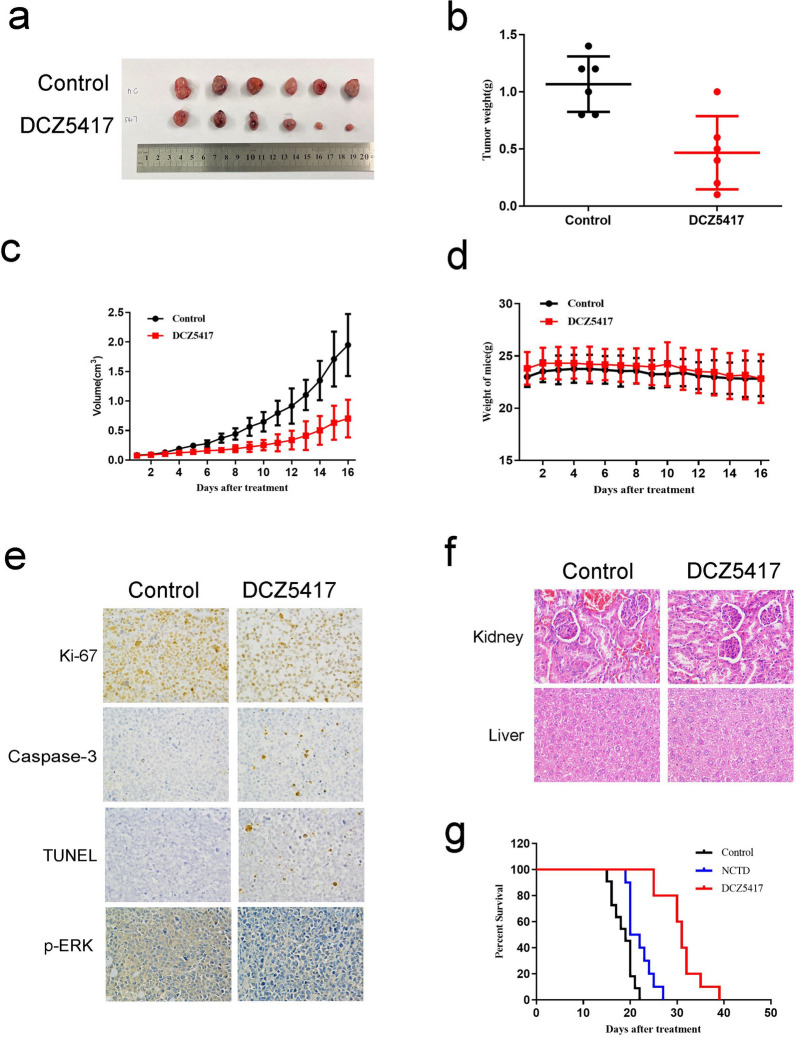
DCZ5417 inhibited tumor growth in vivo. Antitumorigenic effects of DCZ5417 in a xenograft model of MM. **a** H929 cells were injected subcutaneously into mice (n = 6/group) and then mice were treated with or without 15 mg/kg DCZ5417 every day for 16 days. Tumor specimens photographed with a high-definition digital camera. **b** and **c** Tumor sizes were measured every 2 days. Average and SD of the tumor volumes (cm^3^) versus time. **d** Averages and SDs of nude mouse weights versus the time (mean weight SD; 6/group). **e** Ki-67, Caspase-3, TUNEL and p-ERK staining of the control and DCZ5417-treated xenograft tumor tissues (original magnification: ×400). Graphs of the percentage of survival over time (until the tumor volume reached 2000 mm^3^) for the duration of the experiment. “Control” and “DCZ5417” represent mice bearing tumors that were treated with the vehicle or DCZ5417, respectively. **f** H&E staining of the control and DCZ5417-treated liver and kidney tissues in the nude mice (original magnification: ×200). **g** Kaplan–Meier plots of mice treated with the vehicle or DCZ5417 (n = 9/group). Data are presented as the means ± SD of 3 independent experiments. *P < 0.05, **P < 0.01 and ***P < 0.001 versus the control group

An MM xenograft model was then used to investigate the therapeutic potential of DC5417 in vivo. We administered DC5417 or vehicle daily to the mice via an intraperitoneal injection. Compared to that in control mice, the administration of DC5417 significantly reduced tumor growth in immunodeficient mice (Fig. 7a, b). Moreover, compared to those in the vehicle group, the administration of DCZ5417 significantly decreased tumor weights (Fig. 7c). No significant weight differences were observed between DCZ5417 and vehicle administration groups, indicating that DCZ5417 was well tolerated (Fig. 7d). Furthermore, we performed a pharmacodynamic study in which the harvested tumors were analyzed for antiproliferative, apoptotic, and ERK/MAPK signaling-related protein markers. Compared to those in control mice, mice treated with DCZ5417 exhibited decreases in tumor Ki-67 and p-ERK levels. However, compared to that with vehicle treatment, an increase in cleaved caspase-3 and TUNEL staining was observed in mice treated with DCZ5417 (Fig. 7e). To further examine the toxicity of DCZ5417, hematoxylin and eosin (H&E) staining of major organs was performed. There were no significant histological changes in the livers and kidneys of the mice in the two groups (Fig. 7f), indicating that the side effects of DCZ5417 were minimal. To study the effects of DCZ5417 treatment on mouse survival, another MM mouse xenograft model was used. We administered DC5417, NCTD, or vehicle daily to mice via intraperitoneal injection. Significantly, treatment with DCZ5417 prolonged overall survival compared to that in the NCTD-treated animals (Fig. 7g). These findings suggest that DCZ5417 induces potent anti-myeloma responses in mice and prolongs their survival.

## Discussion

DCZ5417 is an available synthetic derivative of NCTD. The present study showed that it possessed lower toxicity, better safety, and greater activity than NCTD against primary MM and MM cell lines. The IC_50_ of DCZ5417 against primary MM cells was in the range of 1–10 mol/L. Moreover, this drug induced significant antitumor activity that was associated with a significant decrease in the tumor burden and prolonged overall survival. Combined treatment with DCZ5417 and conventional anti-MM drugs, specifically melphalan or lenalidomide, induced synergistic anti-MM effects, which provided evidence for the beneficial effects of combination therapy, such as reducing the required concentration and potential side effects.

Although various tumor growth-inhibitory effects of NCTD have been reported, clinical trials on this compound are limited owing to its severe adverse effects [10]. Based on this natural product and our previous study, we optimized the structure of NCTD by changing the oxa-endocyclic skeleton to DCZ5417, an aliphatic endocyclic compound. A ten fold higher inhibitory potency, relative to that of NCTD, based on several MM cell lines was verified, and less acute toxicity and better safety were observed in the DCZ5417 group. Structural optimization efforts have demonstrated the utility of natural product-like analog modifications, and this will become a driving force for preclinical research on NCTD-like derivatives [10, 13].

Virtual docking analysis revealed that DCZ5417 targeted TRIP13. We performed a series of assays, including pull-down, CETSA, and SPR, which provided evidence that DCZ5417 binds to TRIP13. We previously reported that TRIP13 was an oncogene in MM that was related to poor prognosis and identified it as an effective validated target [17, 24]. Gene loss and function assays indicated that DCZ5417 targeted TRIP13. To our knowledge, this is the first study to report DCZ0415 as a TRIP13 inhibitor. In this study, we transformed the oxa-endocyclic skeleton into the novel compound DCZ5417, providing a new direction of transformation and a theoretical basis for clinical practice.

The detailed mechanism by which DCZ5417 targeted TRIP13 was also fully explained in this study. Our results also suggest that TRIP13 acts as an oncogene by activating the MAPK signaling pathway in MM, which requires further investigation. TRIP13 is reportedly involved in checkpoint signaling, DNA break repair and recombination, and chromosome synapsis, leading to cancer initiation and progression [17, 27]. A mechanistic study also showed that TRIP13 regulated cell growth via NF-KB, AKT–mTOR, and Wnt/β-catenin signaling pathways [19, 20, 24]. In the present study, we investigated the involvement of TRIP13 in the ERK/MAPK signaling pathway, and we found that TRIP13 activated ERK/MAPK signaling by interacting with YWHAE. This study thus provides more information on the mechanism of TRIP13-mediated cell growth from the perspective of ERK/MAPK signaling. It is well known that the cytokines IL-6 and IGF-1 are activators of the ERK/MAPK signaling pathway, and they have been used to mimic the microenvironment [28, 29]. In this study, we found that DCZ5417 significantly abrogated the cell proliferation mediated by IL-6 and IGF-1. Our data further demonstrated that DCZ5417 disrupted the interaction between TRIP13 and YWHAE, leading to the inactivation of ERK/MAPK signaling and cell cycle suppression, which explained why DCZ5417 could overcome the protective effect of the microenvironment.

Modern medicine, especially that used for treating tumors, is usually limited by the toxic side effects of single drugs. The effect of treatment cannot be achieved by increasing the dose of a single drug indefinitely, which would be accompanied by marked side effects. Therefore, two drugs with different side effects are selected for two-drug combination therapy at lower doses [30]. Thus, we examined the synergistic effects of DCZ5417 and other anti-myeloma agents, including melphalan and lenalidomide. Interestingly, DCZ5417 significantly enhanced the cytotoxic effects of melphalan and lenalidomide in both MM cell lines and primary cells from myeloma patients, suggesting that DCZ5417 is a promising anti-myeloma drug.

## Conclusions

Here, we provided a novel NCTD derivative, named DCZ5417, with fewer side effects that can suppress MM progression in vitro, in vivo, and in primary cells from drug-resistant patients. We also suggest that DCZ5417 suppresses cell proliferation by targeting TRIP13, destroying the TRIP13/YWHAE complex, and inhibiting the ERK/MAPK signaling axis. This study provides a new and effective therapeutic strategy for the treatment of MM.

### Supplementary Information

**Additional file 1: Figure S1. **Development for a better effect and lower toxicity derivative of NCTD. **Figure S2.** DCZ5417 inhibits multiple myeloma.** Figure S3.** DCZ5417 inhibits MAPK signaling pathway.

## Data Availability

The datasets supporting the conclusions of this article are included in this published article (and its Additional files).

## Abbreviations

MM: Multiple myeloma
NCTD: Norcantharidin
TRIP13: Thyroid hormone receptor interacting protein 13
ATCC: American Type Culture Collection
SPR: Surface plasmon resonance
CETSA: Cellular thermal shift assay
PBMCs: Peripheral blood mononuclear cells
PCL: Plasma cell leukemia

## References

[1] Kuiper R, Broyl A, de Knegt Y, van Vliet MH, van Beers EH, van der Holt B, el Jarari L, Mulligan G, Gregory W, Morgan G (2012). A gene expression signature for high-risk multiple myeloma. Leukemia.

[2] Kumar SK, Rajkumar V, Kyle RA, van Duin M, Sonneveld P, Mateos MV, Gay F, Anderson KC (2017). Multiple myeloma. Nat Rev Dis Primers.

[3] Waxman AJ, Mink PJ, Devesa SS, Anderson WF, Weiss BM, Kristinsson SY, McGlynn KA, Landgren O (2010). Racial disparities in incidence and outcome in multiple myeloma: a population-based study. Blood.

[4] Huang SY, Yao M, Tang JL, Lee WC, Tsay W, Cheng AL, Wang CH, Chen YC, Shen MC, Tien HF (2007). Epidemiology of multiple myeloma in Taiwan: increasing incidence for the past 25 years and higher prevalence of extramedullary myeloma in patients younger than 55 years. Cancer.

[5] Drayson M, Tang LX, Drew R, Mead GP, Carr-Smith H, Bradwell AR (2001). Serum free light-chain measurements for identifying and monitoring patients with nonsecretory multiple myeloma. Blood.

[6] Rajkumar SV, Dimopoulos MA, Palumbo A, Blade J, Merlini G, Mateos MV, Kumar S, Hillengass J, Kastritis E, Richardson P (2014). International Myeloma Working Group updated criteria for the diagnosis of multiple myeloma. Lancet Oncol.

[7] Laubach J, Richardson P, Anderson K (2011). Multiple myeloma. Annu Rev Med.

[8] Palumbo A, Anderson K (2011). Multiple myeloma. N Engl J Med.

[9] Mahindra A, Laubach J, Raje N, Munshi N, Richardson PG, Anderson K (2012). Latest advances and current challenges in the treatment of multiple myeloma. Nat Rev Clin Oncol.

[10] Zhai BT, Sun J, Shi YJ, Zhang XF, Zou JB, Cheng JX, Fan Y, Guo DY, Tian H (2022). Review targeted drug delivery systems for norcantharidin in cancer therapy. J Nanobiotechnology.

[11] Hsieh CH, Chao KS, Liao HF, Chen YJ (2013). Norcantharidin, derivative of cantharidin, for cancer stem cells. Evid Based Complement Alternat Med.

[12] Liu Z, Li B, Cao M, Jiang J (2021). Norcantharidin triggers apoptotic cell death in non-small cell lung cancer via a mitophagy-mediated autophagy pathway. Ann Transl Med.

[13] Zheng J, Du W, Song LJ, Zhang R, Sun LG, Chen FG, Wei XT (2014). Norcantharidin induces growth inhibition and apoptosis of glioma cells by blocking the Raf/MEK/ERK pathway. World J Surg Oncol.

[14] Lu S, Qian J, Guo M, Gu C, Yang Y (2019). Insights into a crucial role of TRIP13 in human cancer. Comput Struct Biotechnol J.

[15] Miniowitz-Shemtov S, Eytan E, Kaisari S, Sitry-Shevah D, Hershko A (2015). Mode of interaction of TRIP13 AAA-ATPase with the Mad2-binding protein p31comet and with mitotic checkpoint complexes. Proc Natl Acad Sci U S A.

[16] Ye Q, Kim DH, Dereli I, Rosenberg SC, Hagemann G, Herzog F, Toth A, Cleveland DW, Corbett KD (2017). The AAA+ ATPase TRIP13 remodels HORMA domains through N-terminal engagement and unfolding. EMBO J.

[17] Tao Y, Yang G, Yang H, Song D, Hu L, Xie B, Wang H, Gao L, Gao M, Xu H (2017). TRIP13 impairs mitotic checkpoint surveillance and is associated with poor prognosis in multiple myeloma. Oncotarget.

[18] Zhang G, Zhu Q, Fu G, Hou J, Hu X, Cao J, Peng W, Wang X, Chen F, Cui H (2019). TRIP13 promotes the cell proliferation, migration and invasion of glioblastoma through the FBXW7/c-MYC axis. Br J Cancer.

[19] Zhu MX, Wei CY, Zhang PF, Gao DM, Chen J, Zhao Y, Dong SS, Liu BB (2019). Elevated TRIP13 drives the AKT/mTOR pathway to induce the progression of hepatocellular carcinoma via interacting with ACTN4. J Exp Clin Cancer Res.

[20] Liu X, Shen X, Zhang J (2021). TRIP13 exerts a cancer-promoting role in cervical cancer by enhancing Wnt/beta-catenin signaling via ACTN4. Environ Toxicol.

[21] Zhou XY, Shu XM (2019). TRIP13 promotes proliferation and invasion of epithelial ovarian cancer cells through Notch signaling pathway. Eur Rev Med Pharmacol Sci.

[22] Deriano L, Roth DB (2013). Modernizing the nonhomologous end-joining repertoire: alternative and classical NHEJ share the stage. Annu Rev Genet.

[23] Li C, Xia J, Franqui-Machin R, Chen F, He Y, Ashby TC, Teng F, Xu H, Liu D, Gai D (2021). TRIP13 modulates protein deubiquitination and accelerates tumor development and progression of B cell malignancies. J Clin Invest.

[24] Wang Y, Huang J, Li B, Xue H, Tricot G, Hu L, Xu Z, Sun X, Chang S, Gao L (2020). A small-molecule inhibitor targeting TRIP13 suppresses multiple myeloma progression. Cancer Res.

[25] Xie Y, Wang Y, Xu Z, Lu Y, Song D, Gao L, Yu D, Li B, Chen G, Zhang H (2022). Preclinical validation and phase I trial of 4-hydroxysalicylanilide, targeting ribonucleotide reductase mediated dNTP synthesis in multiple myeloma. J Biomed Sci.

[26] Esma F, Salvini M, Troia R, Boccadoro M, Larocca A, Pautasso C (2017). Melphalan hydrochloride for the treatment of multiple myeloma. Expert Opin Pharmacother.

[27] Roig I, Dowdle JA, Toth A, de Rooij DG, Jasin M, Keeney S (2010). Mouse TRIP13/PCH2 is required for recombination and normal higher-order chromosome structure during meiosis. PLoS Genet.

[28] Manier S, Kawano Y, Bianchi G, Roccaro AM, Ghobrial IM (2016). Cell autonomous and microenvironmental regulation of tumor progression in precursor states of multiple myeloma. Curr Opin Hematol.

[29] Liu J, Hideshima T, Xing L, Wang S, Zhou W, Samur MK, Sewastianik T, Ogiya D, An G, Gao S (2021). ERK signaling mediates resistance to immunomodulatory drugs in the bone marrow microenvironment. Sci Adv.

[30] Adnane F, El-Zayat E, Fahmy HM (2022). The combinational application of photodynamic therapy and nanotechnology in skin cancer treatment: A review. Tissue Cell.

