# Supplementary material for: The novel norcantharidin derivative DCZ5417 suppresses multiple myeloma progression by targeting the TRIP13–MAPK–YWHAE signaling pathway
Source: J Transl Med. 2023 Nov 27;21:858. doi: 10.1186/s12967-023-04739-7 (PMC10680230; doi:10.1186/s12967-023-04739-7)
Supplement: Supplementary file 1 — Additional file 1: Figure S1. Development for a better effect and lower toxicity derivative of NCTD. Figure S2. DCZ5417 inhibits multiple myeloma. Figure S3. DCZ5417 inhibits MAPK signaling pathway. [file 12967_2023_4739_MOESM1_ESM.pdf]

Figure S1

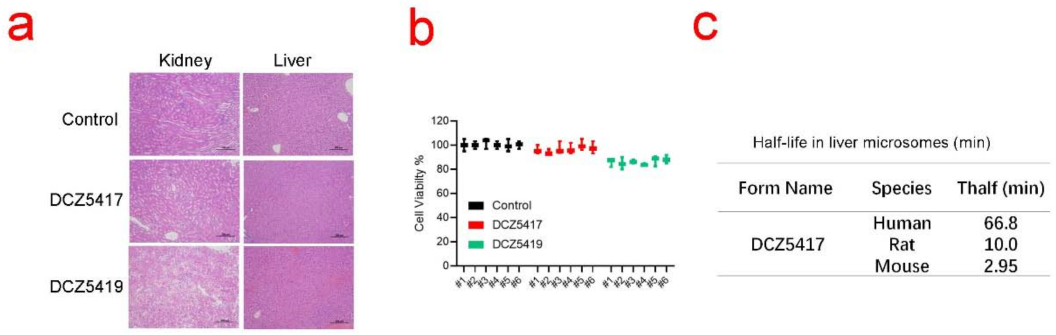

Figure S1 Development for a better effect and lower toxicity derivative of NCTD

(a) Mice were treated with or without DCZ5417, DCZ5419 or vehicle, respectively (6/group). H&E staining of the control, DCZ5419 and DCZ5417-treated liver and kidney tissues. (b) Normal PBMCs from healthy donors (PBMCs#1–PBMCs#6) were treated with 20μmol/L DCZ5417 or DCZ5419 for 48 hours and then cell viability was analyzed. (c)The metabolic stability of DCZ5417 in human, rat and mouse liver microsomes was determined.

Figure S2

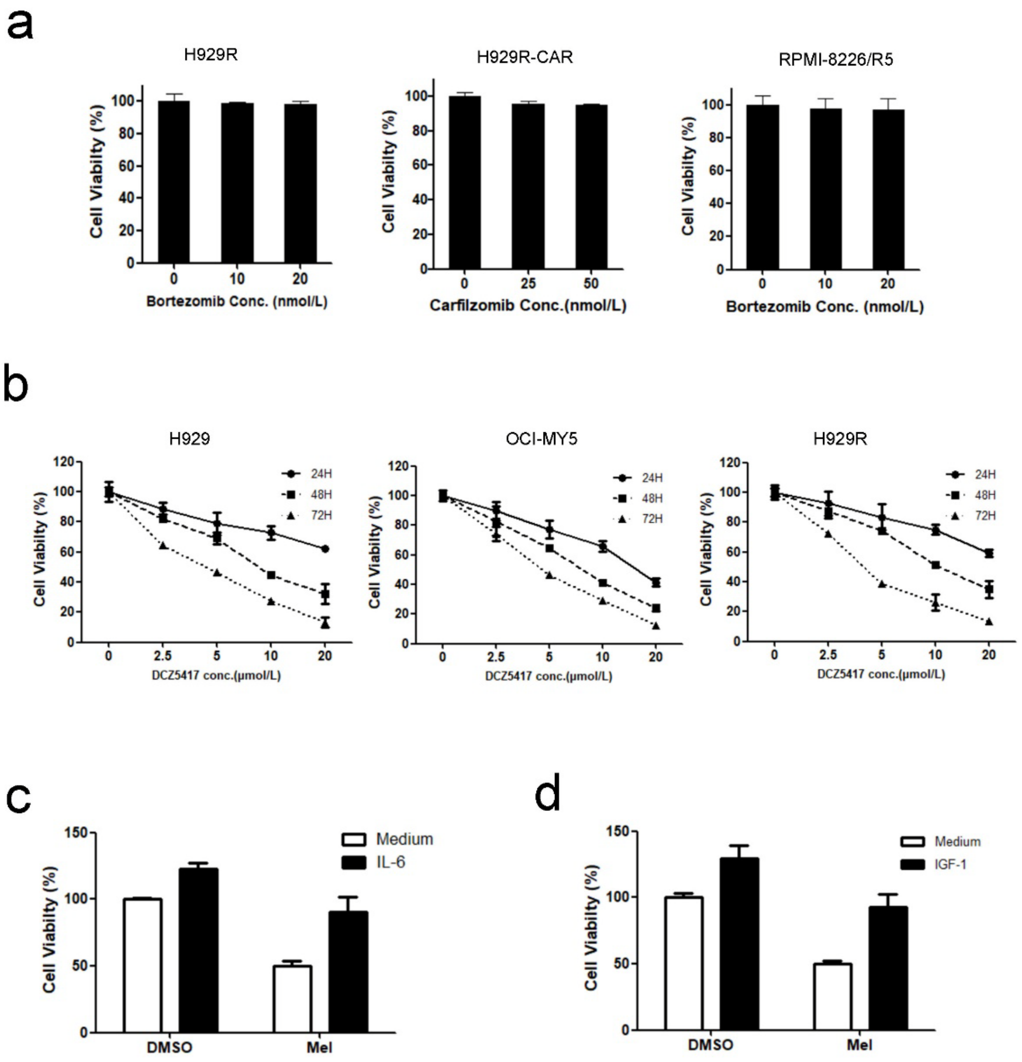

Figure S2 DCZ5417 inhibits multiple myeloma.

(a)Indicated MM cell lines were respectively treated with bortezomib or carfilzomib at the indicated concentrations for 72h. Then the cell viability was determined by CCK-8 assay. (b) Indicated MM cell lines were treated with vehicle or DCZ5417 at the indicated concentrations for 24h, 48h and 72h. Then the cell viability was determined by CCK-8 assay. Cell viability data are presented as the means of 3 independent experiments. (c and d) Activity of melphalan against H929 cells cultured in the presence or absence of IL-6 or IGF-1 for 48 hours. Error bars, SD. The result is expressed as means±SD of three independent experiments.

Figure S3

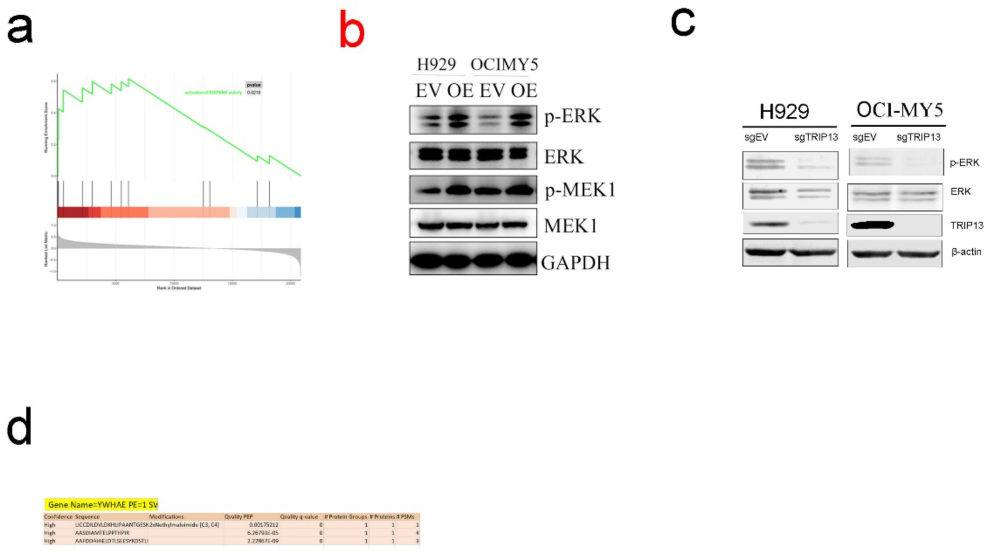

Confidence	Sequence	Modifications	Qvality PEP	Qvality q-value	# Protein Groups	# Proteins	# PSMs
High	LCCDILDVLDKHLIPAANTGESK	2xNethylmaleimide [C3; C4]	0.00175212	0	1	1	1
High	AASDIAMTELPPTHPIR		6.26793E-05	0	1	1	4
High	AAFDDAIAELDTLSEESYKDSTLI		2.22867E-09	0	1	1	3

Figure S3 DCZ5417 inhibits MAPK signaling pathway.

(a)Gene Set Enrichment (GSEA) analysis was conducted according to publicly available MM patient data sets. An enrichment plot for MAPK pathway in cells. (b) Immunoblotting analysis was conducted to test protein level of p-ERK/ERK and p-MEK/MEK in TRIP13-OE and EV cells. (c) Immunoblotting analysis was conducted to test protein level of pERK/ERK in sgTRIP13 and sgControl cells. SgControl represents nontarget scramble–transfected cells. TRIP13 sgRNA represents TRIP13-silenced cells. (d) LC–MS/MS were performed in TRIP13 OE and Vector cells. Vector represents nontarget scramble–transfected cells. TRIP13 OE represents overexpression of TRIP13 in cells.
